# Effect of Thermomechanical Treatment on Structure and Functional Fatigue Characteristics of Biodegradable Fe-30Mn-5Si (wt %) Shape Memory Alloy

**DOI:** 10.3390/ma14123327

**Published:** 2021-06-16

**Authors:** Sergey Prokoshkin, Yury Pustov, Yulia Zhukova, Pulat Kadirov, Maria Karavaeva, Alexey Prosviryakov, Sergey Dubinskiy

**Affiliations:** 1 Metal Forming Department, National University of Science and Technology “MISiS”, 119049 Moscow, Russia; prokoshkin@tmo.misis.ru (S.P.); dubinskiy.sm@misis.ru (S.D.); 2 Department of Steel Metallurgy, New Production Technologies and Protection of Metals, National University of Science and Technology “MISiS”, 119049 Moscow, Russia; pustov@misis.ru; 3 Center of Nanomaterials and Nanotechnologies, National University of Science and Technology “MISiS”, 119049 Moscow, Russia; pulat_1993-2009@mail.ru (P.K.); karavaeva.ma@yandex.ru (M.K.); 4 Ultrafine-Grained Metallic Materials Laboratory, National University of Science and Technology “MISiS”, 119049 Moscow, Russia; pro.alex@mail.ru

**Keywords:** iron alloys, shape memory alloys, biodegradable metals, functional properties, thermomechanical treatment, mechanical properties, corrosion and electrochemical behavior, corrosion fatigue

## Abstract

The Fe-Mn-Si shape memory alloys are considered promising materials for the biodegradable bone implant application since their functional properties can be optimized to combine bioresorbability with biomechanical and biochemical compatibility with bone tissue. The present study focuses on the fatigue and corrosion fatigue behavior of the thermomechanically treated Fe-30Mn-5Si (wt %) alloy compared to the conventionally quenched alloy because this important functionality aspect has not been previously studied. Hot-rolled and water-cooled, cold-rolled and annealed, and conventionally quenched alloy samples were characterized by X-ray diffraction, transmission electron microscopy, tensile fatigue testing in air atmosphere, and bending corrosion fatigue testing in Hanks’ solution. It is shown that hot rolling at 800 °C results in the longest fatigue life of the alloy both in air and in Hanks’ solution. This advantage results from the formation of a dynamically recrystallized *γ*-phase grain structure with a well-developed dislocation substructure. Another important finding is the experimental verification of Young’s modulus anomalous temperature dependence for the studied alloy system, its minimum at a human body temperature, and corresponding improvement of the biomechanical compatibility. The idea was realized by lowering *M_s_* temperature down to the body temperature after hot rolling at 800 °C.

## 1. Introduction

In the last decade, biodegradable iron alloys, particularly the Fe-Mn-based system, have been considered an alternative to conventional biomedical alloys based on titanium, cobalt, stainless steels, and other metallic materials used in dentistry, orthopedics, and surgery [1,2,3,4,5,6,7,8]. These materials combine the necessary functional properties (high mechanical properties and appropriate in vitro and in vivo biocompatibility) and, in contrast to traditional alloys, exhibit the ability to self-dissolve in the osteogenesis process [5,9,10,11,12,13]. However, pure iron dissolves at a low rate (0.10 mm/year), and even the introduction of electrochemically active manganese into the composition without additional impact on the alloy structure only slightly increases the biodegradation rate (Fe-30Mn, 0.11 mm/year) [14] in Hanks’ solution [15], which is insufficient for the complete elimination of the alloy components from the body after the completion of the damaged tissue regeneration process [1,5]. However, the biodegradation rate of the Fe-30Mn alloy can be increased several times after the homogenization of its structure by conducting isothermal annealing for 1 h followed by quenching in water [16,17].

In this regard, Fe-Mn alloys doped with silicon are of great practical interest. At a certain manganese content (28–33 wt %), the introduction of 4–6 wt % Si to the alloy composition leads not only to the hardening of the alloys [18] but also facilitates the realization of the shape memory effect [17,19,20]. Moreover, the heat treatment of the Fe-30Mn-5Si alloy [17], which ensured the stabilization of the martensitic structure, made it possible to significantly reduce the martensitic start (*M_s_*) and finish (*M_f_*) temperatures: 60 °C and below 60 °C, respectively, in comparison with the Fe-(23-26)Mn-5Si. According to [17], this is due to the crystal lattice deformation caused by high Mn content, leading to a slowdown in the *γ—ε* transformation and/or a high austenite volume fraction.

Currently, the strategy to achieve the optimal biodegradation rate of Fe-Mn-based alloys comprises of the following two approaches reviewed in [5,21]: chemical (alloying) and technological (production processes and post-production processing).

Alloying of iron-based alloys is carried out taking into account the following basic approaches [22]: (1) alloying with more electronegative elements (Mn, Co, Al, W, Sn, B, C, and S), providing an increase in the electrochemical activity of the alloys; (2) the addition of more noble alloying elements or nanoscale particles that form microgalvanic pairs with an iron matrix, in which iron is an anodic structural component; in particular, the introduction of only 1 wt % Pd into the Fe-21Mn-0.7C alloy resulted in a twofold increase in the corrosion rate (0.21 mm/year).

The technological approach to increasing the biodegradation rate is implemented by (1) controlling the structure of the bulk material through thermal or thermomechanical treatment, (2) producing porous structures, (3) applying novel methods of metal forming treatment, for example, accumulative cryo-rolling at the temperature of liquid nitrogen and subsequent annealing, severe plastic deformation methods, etc.; (4) modification of the alloy surface. The main principle of increasing the degradation rate in the framework of this approach is the formation of a multiphase and/or more defective microstructure (for the methods of thermomechanical treatment), an increase in the specific surface area (in the case of creating porous structures) [21].

Apart from increasing the dissolution rate of the alloys, it deems crucial to achieving the combination of all functional properties: the degradation rate in a corrosive biological environment and mechanical characteristics (elastic limit, ultimate strength, etc.). In the case of intraosseous implant material, special attention should be paid to the biomechanical compatibility issue [9]—the similarity of the implanted material mechanical properties and those of the surrounding bone tissue, principally, Young’s modulus value. It is known that Young’s modulus decrease near the *M_s_* temperature is common for shape memory alloys (SMAs) [23]; therefore, these biodegradable alloys’ processing regimes should ensure that *M_s_* is close to the human body temperature.

Studies of Fe-(23...30) Mn-5Si alloys (wt %) described in [16,17,24] showed the possibility of achieving optimal characteristics by implementing certain regimes of thermal and thermomechanical treatment leading to the rise of biodegradation rate from 0.4 to 0.8 mm/year, the decrease in the elastic modulus from 120 to 97 GPa, and a decrease *M_s_* temperature from 70 to 38 °C. The best characteristics were obtained for the Fe-30Mn-5Si alloy [24].

Of particular interest is the study of the Fe-Mn-Si alloys’ functional fatigue and corrosion fatigue properties since they are subjected to cyclic dynamic loading in the process of their performance as biodegradable implants. The accumulation of plastic deformation resulting from the action of cyclic mechanical stress can lead to the initiation and propagation of fatigue cracks in the regions of stress concentration. In [25,26,27,28,29,30], the fatigue behavior of the Fe-Mn-Si engineering alloys was studied at relatively small deformations; it is shown that the mechanocycling process is accompanied by an initial hardening followed by softening and secondary cyclic hardening.

It was shown [31] that mechanocycling leads to a decrease in the reactive stress value, which is associated with the relaxation process during martensitic transformation during the dynamic load action. However, studies reported in [25,26,27,28,29,30,31] describe the fatigue behavior of Fe-Mn-Si alloys exclusively as engineering materials. The selected deformation modes, the type, and the magnitude of the applied load differ significantly from those experienced by these alloys as bone implant materials [9]. Therefore, the results of these studies addressing the influence of cyclic dynamic load on the fatigue characteristics of these alloys cannot be used unambiguously to assess their biomechanical compatibility. Moreover, the destructive effect of the load is amplified by the corrosive biological environment, i.e., at a given stress level, fracture occurs with a smaller number of cycles than in the absence of a corrosive medium. The importance of considering the superposition effect of these factors was pointed out in [14], where the comprehensive study of the Fe-30Mn-6Si alloy was carried out, and a recommendation was given to use it as a promising biodegradable material with shape memory property.

Given the above and based on the results obtained in [16,17,24], the main goal of this work is to study the Fe-30Mn-5Si alloy fatigue behavior features in the initial state (homogenized and quenched) and after thermomechanical treatment (TMT) using mechanical cyclic tests in air and Hanks’ solution, the study of Young’s modulus temperature dependence in the temperature range above and close to the *M_s_* to verify the existence of lattice softening effect, which is characteristic of shape memory alloys, and the role of martensitic transformation in corrosion fatigue failure of the alloy.

## 2. Materials and Methods

The investigated alloy Fe-30Mn-5Si (wt %) was obtained from high-purity raw materials by vacuum-arc remelting with a non-consumable tungsten electrode, with preliminary remelting of the getter (titanium); this technique has previously been shown as effective for obtaining high-purity materials, including those for biomedical purposes [32]. In order to eliminate dendritic segregation, homogenization annealing was carried out at 900 °C for 60 min, followed by water quenching [16]; hereinafter, this treatment regime is referred to as reference heat treatment (RHT). The chemical composition and its uniformity throughout the ingot were measured by energy-dispersive X-ray spectroscopy using a JSM-6480 LV scanning microscope (JEOL, Eching b. München, Germany) in no less than ten points [24].

The choice of TMT regimes aimed at creating various structural states of the material based on [24], as follows: a developed dislocation substructure or a fine-grained recrystallized structure during (a) hot deformation at 600 and 800 °C (true deformation strain *e* = 0.3), hereinafter designated as HR_600_ and HR_800_ regimes, respectively; (b) cold deformation (*e* = 0.3) with post-deformation annealing at 500 and 600 °C for 30 min, hereinafter designated as CR_500_ and CR_600_ regimes, respectively. Samples for further preparation were cut by an electrical discharge machine.

Hot rolling of specimens (size 16 mm × 80 mm × 22 mm) was carried out on a laboratory Rolling Mill Duo in 4–5 passes, with intermediate heating at a deformation temperature for 5 min after each pass. Upon reaching the desired size, the samples were cooled in water. Cold rolling was carried out on a laboratory Rolling Mill Duo at room temperature in 4–5 passes, followed by post-deformation annealing and subsequent water cooling.

A transmission electron microscopy (TEM) study of the thermomechanically treated Fe-30Mn-5Si alloy structure was carried out on a JEM 2100 transmission electron microscope (JEOL, Eching b. München, Germany). Samples 10 mm × 10 mm × 0.3 mm in size were mechanically thinned to 0.1 mm and then thinned by electrolytic polishing in a Struers Electrolyte A2 mixture.

Cyclic fatigue tensile tests to failure were performed on an MTS MiniBionix 858 setup (MTS Systems, Eden Prairie, MN, USA) with a strain rate of 0.02 s^–1^ on specimens 70 mm × 1.5 mm × 1 mm in size. Fatigue behavior was studied at room temperature by mechanical cycling according to the “loading–unloading” scheme until failure with a maximum deformation of 0.5% per cycle. The elastic strain of the bone tissue is generally 0.2%. In this study, the chosen maximum strain in the cycle 2.5 times exceeded this value to simulate the extreme loading conditions. In each cycle, according to the “loading–unloading” diagram, Young’s modulus *E* was determined from the slope of the load branch, the “dislocation” yield stress *σ_0.05_*, the residual strain in the cycle *ε_f_*, the accumulated strain after each cycle *ε_acc_*, and the number of cycles to failure *N_max_*.

The temperature dependence of Young’s modulus was determined from stress–strain diagrams obtained during specimen tension experiment on a Zwick Z250 universal testing machine (ZwickRoell, Ulm, Germany) using an EXMACRO automatic extensometer (ZwickRoell, Ulm, Germany) and a replaceable load cell with a maximum force of 10 kN. Heating/cooling was carried out in a heat chamber providing access to the sample of high-temperature probes of the strain gauge. The extensometer measuring distance was 15 mm. The sample was stretched to an elongation of 0.05% (the area of elastic strain was selected to avoid strain hardening of the alloy). The tensile rate was 1 mm/min. The sample was fixed using wedge-screw grips placed in a heat chamber. The grips were preheated for 90 min. The sample was held at a given temperature for 30 min. Young’s modulus was calculated by regression using the testXpert III program (ZwickRoell, Ulm, Germany), and by the slope of a straight line coinciding with the initial linear section of the load loop deformation diagram to the abscissa axis. In accordance with the DSC data obtained in [24], the following temperatures for carrying out mechanical tensile tests were selected: 250, 200, 150, 100, and 50 °C, and room temperature. Tests for all experimental temperatures included loading to a strain of 0.05%. In this case, each sample was first heated at 300 °C, and then cooled to the test temperature. Thus, in the initial state before stretching in the temperature range of 50–250 °C, the alloy was in the austenitic state, and at room temperature it contained a small amount of martensite.

The change in the phase composition during mechanocycling of the HR_800_-treated alloy was studied after 0 (initial state), 1, 10, 30, and 50 loading cycles by X-ray diffraction (XRD) analysis on a DRON-3.0 diffractometer (Bourevestnik, Saint-Petersburg, Russia) using CuKα-radiation at room temperature. The *2θ* angle range was from 30 to 100 degrees with a step of 0.3 and an exposure of 3 s.

**Figure 1:**
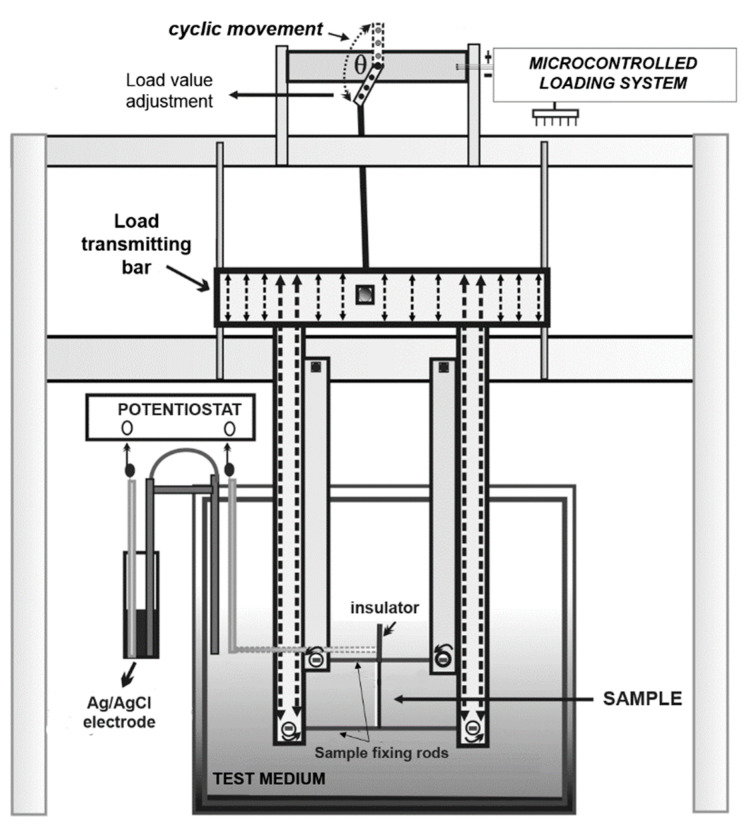
The principal design of the experimental setup for corrosion fatigue testing under cyclic loading [33,34,35].

Corrosion fatigue testing of alloy specimens for bending was carried out in Hanks’ solution [15] at 37 °C on an experimental setup that provides registration of the electrode potential (open circuit potential, OCP) of the alloy by an electronic potentiostat IPC-Pro MF (Volta Co. Ltd., Saint-Petersburg, Russia) at a given cyclic loading mode (frequency and amplitude) [33,34,35]; the size of the samples was 70 mm × 3 mm × 0.5 mm, at least five samples for each test. The temperature (37 ± 1) °С in the working chamber was maintained using a TW-2 Elmi laboratory thermostat (Elmi, Riga, Latvia). The principal design of the test setup is shown in Figure 1. Before switching on the load, the samples were kept in the solution until a constant OCP value was established, which indicated that a stationary corrosion regime was achieved. The failure of the sample after a certain number of cycles was registered by the rupture of the electrical circuit between the sample and the silver chloride reference electrode resulting in the OCP drop.

The elemental composition and chemical state of corrosion products were determined using a PHI5000VersaProbeII X-ray photoelectron spectrometer (XPS) (Physical Electronics Inc., Chanhassen, MN, USA) using monochromatic Al Kα radiation (*hν* = 1486.6 eV) with a power of 50 W. Atomic concentrations were calculated from the survey spectra by the method of relative elemental sensitivity factors.

The abbreviations and symbols introduced in the manuscript text are listed in Appendix A.

## 3. Results and Discussion

### 3.1. Microstructure of Thermally and Thermomechanically Treated Fe-30Mn-5Si Alloy

Following previous studies of the grain structure of Fe-30Mn-5Si alloy after the RHT and TMTs using optical microscopy and X-ray diffraction analysis [16,24], the *γ*–austenite grain size is about 500 µm after RHT. The hot deformation at 600 °С (HR_600_) does not lead to grain refinement but results in elongation along the rolling direction, indicating the absence of dynamic recrystallization. Raising the deformation temperature to 800 °С (HR_800_) led to grain refinement down to about 100 µm and equiaxed grain shape due to the dynamic recrystallization. The cold rolling with post-deformation annealing at 500 and 600 °С (CR_600_ and CR_600_) did not lead to grain refinement. The phase state of the Fe-30Mn-5Si alloy is characterized by the presence of two phases, γ–austenite and ε–martensite. Besides, it has been shown that the TMTs lead to lowering the martensitic transformation temperature range towards room temperature, decrease in the ε-martensite amount, and significant broadening of the X-ray diffraction lines as compared to the RHT [16,24].

**Figure 2:**
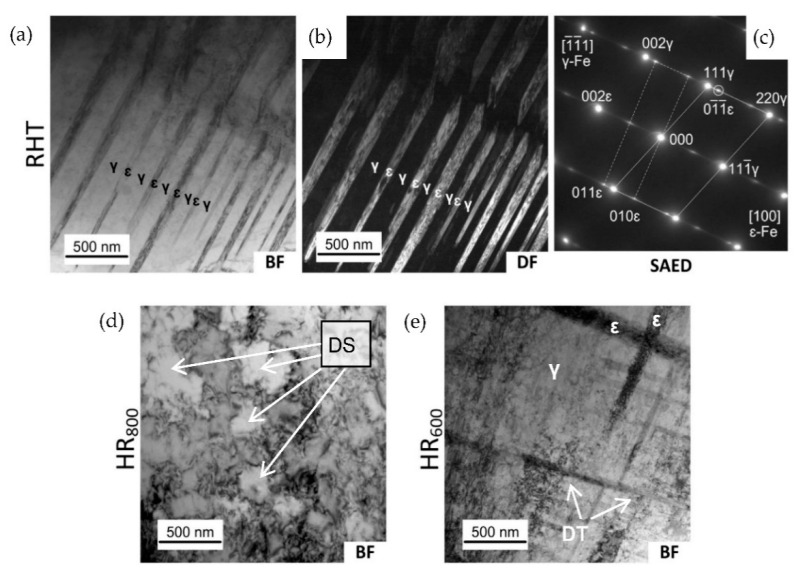
Structure of the Fe-30Mn-5Si alloy: (**a**–**c**) RHT; (**d**) HR_800_; (**e**) HR_600_. Transmission electron microscopy. Bright field (BF) and dark field (DF) images, and SAED pattern. DF is taken from 0$\bar{1}\bar{1}$ reflex circled in (**c**). DS—dislocation cells; DT—dislocation tangles.

To clarify the peculiarities of the dislocation substructure in the RHT state and after various treatments, a TEM study was performed after RHT, HR_600_, and HR_800_ regimes. Corresponding bright field (BF) and dark field (DF) images are presented in Figure 2. The bright field image of the structure formed after RHT shows a colony of parallel elongated dark crystals in a matrix that did not contain a high dislocation density (Figure 2a). The corresponding selected area electron diffraction (SAED) pattern consists of FCC γ-austenite matrix reflexes belonging to the <100>*γ* zone axis and some reflexes of HCP ε-martensite (Figure 2b). The DF image of the same area presented in Figure 2c was taken from the 0$\bar{1}\bar{1}$*ε* reflex shows that the elongated crystals became bright, and thus, these crystals represent ε-martensite.

Examples of the structure formed after HR_800_ are presented in Figure 2d, which shows a well-developed dislocation substructure of the austenite, which resembles a cellular substructure. The observed dislocation substructure is an obvious result of the austenite dynamic recrystallization during hot deformation [16,24]. In this case, newly formed recrystallized austenite grains are saturated by dislocations in the course of further deformation [36].

The structure formed after HR_600_ is presented in Figure 2e, which shows a well-developed dislocation substructure of the austenite, and the dislocation density is much higher than after HR_800_. The dislocation tangles and cells are observed, while a typical polygonized dislocation substructure consisting of perfect subgrains does not form. As the dynamic recrystallization does not occur, in this case, the observed dislocation substructure was introduced in initial grains during deformation.

### 3.2. Tensile Fatigue Test in Air

**Figure 3:**
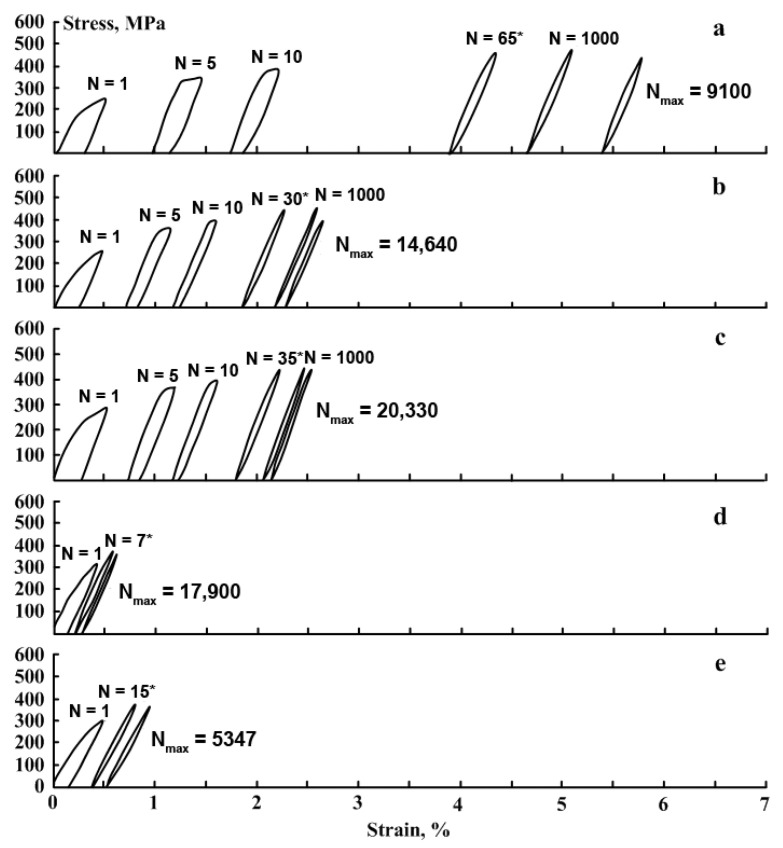
Stress-strain diagrams of the Fe-30Mn-5Si alloy during mechanical cycling (*N* is the number of a cycle) after different treatments: (**a**)-RHT, (**b**)-HR_600_, (**c**)-HR_800_, (**d**)-CR_500_, (**e**)-CR_600_ (*—the beginning of the loop closure).

**Figure 4:**
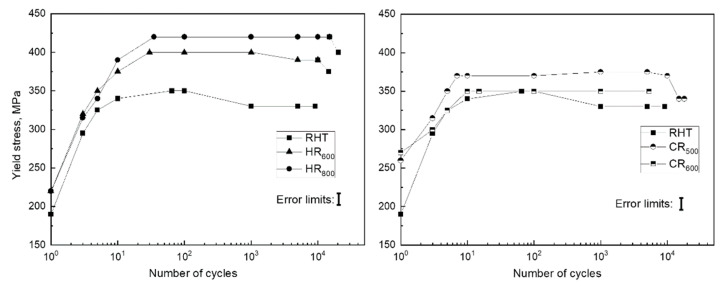
Evolution of the dislocation yield stress with the increasing number of cycles for the Fe-30Mn-5Si alloy after various treatment regimes.

The Fe-30Mn-5Si alloy in the initial state (RHT) and after TMT was subjected to fatigue tests in air in order to study the evolution of the dislocation yield stress, residual strain in each cycle, Young’s modulus, and to determine the total number of cycles to failure and the total accumulated deformation after the failure. Stress–strain diagrams of the Fe-30Mn-5Si alloy during mechanical cycling are shown in Figure 3; the dislocation yield stress values derived from these data are given in Figure 4.

The yield stress of the conventionally treated (RHT) Fe-30Mn-5Si SMA in our work (190 MPa) is comparable with that of the Fe-30Mn-6Si alloy (180 MPa) [3]. The same parameter for thermomechanically treated Fe-30Mn-5Si SMA is not available from literature; however, it amounts to 242 MPa for a hot-forged Fe-30Mn alloy [37], which is comparable with our data for the hot-rolled Fe-30Mn-5Si (220 MPa).

It can be seen from Figure 3 and Figure 4 that for all studied regimes, the values of the dislocation yield stress increase with the growing number of cycles. The values of the dislocation yield stress for different regimes increase within the following limits: RHT-200–330 MPa, HR_600_-200–400 MPa, HR_800_-200–400 MPa, CR_500_-220–375 MPa, and CR_600_-230–360 MPa. Therefore, in the first cycle, the dislocation yield stress after RHT and HR_600_ and HR_800_ does not differ. It takes place because, in the case of RHT, the alloy contains a more significant amount of *ε*-martensite compared to TMT [24], which compensates for the absence of strain hardening after RHT.

An increase in the dislocation yield stress is associated with structural and substructural hardening during mechanocycling due to an increase in the dislocation density and, as shown below, an increase of *ε*-martensite amount in loading half-cycles due to the *γ→ε* phase transformation under stress. As shown in Figure 4, for all investigated regimes, the values of the dislocation yield stress after a certain number of cycles reach constant levels in the range from 350 to 400 MPa. The reason for this is that upon reaching a certain number of cycles (for RHT-65 cycles, HR_600_-30 cycles, HR_800_-35 cycles, CR_500_-7 cycles, and CR_600_-15 cycles), the value of the yield stress rises above the value of the stress achieved at 0.5% strain. This is confirmed by the closure of the loops in the loading–unloading diagrams (Figure 3).

It should be noted that the value of the dislocation yield stress of the thermomechanically treated alloy (HR and CR regimes) was significantly higher than for the initial state (RHT) for each cycle. This indicates a more hardened state of the alloys after TMT due to the developed dislocation substructure formed by the dynamic recovery and polygonization (HR_600_ regime) and dynamic recrystallization (HR_800_ regime), and static polygonization for the CR_500_ and CR_600_ regimes [24]. Since the values of the dislocation yield stress for the CR regimes are somewhat lower than for the HR ones, it can be concluded that the static softening during post-deformation annealing at 500 and 600 °C surpassed the dynamic hardening during hot deformation at 600–800 °C.

**Figure 5:**
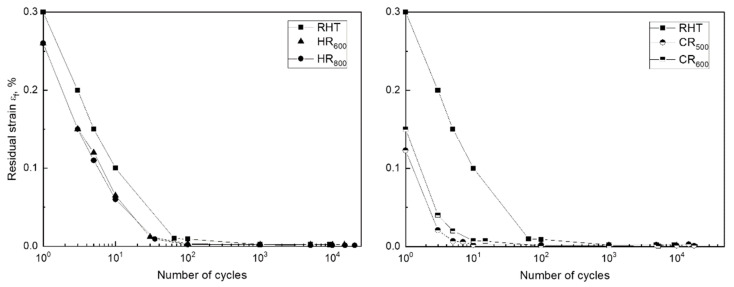
Evolution of the residual strain in the cycle of the Fe-30Mn-5Si alloy after various treatment regimes. The error limits do not exceed the size of symbols.

**Figure 6:**
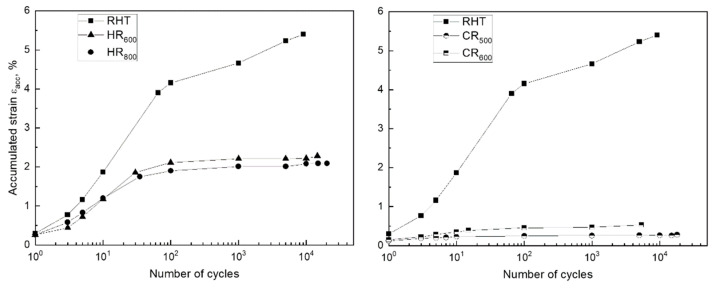
Evolution of the accumulated strain with an increase in the number of cycles for the Fe-30Mn-5Si alloy after various treatment regimes. The error limits do not exceed the size of symbols.

The obtained stress–strain diagrams were used to determine the residual strain *ε_f_* in each cycle and the total accumulated strain *ε_acc_* for the Fe-30Mn-5Si alloy in the initial state and after TMT; the obtained dependencies are shown in Figure 5 and Figure 6, respectively. The residual strain in the first cycle was the greatest after RHT, it was smaller after HR_800_ and HR_600_, and it was the smallest after CR_500_ and CR_600_. Figure 5 shows that for all processing modes, a decrease in the residual strain value is observed in each cycle during mechanocycling; this process occurred more slowly after RHT than after TMT.

A decrease in residual strain in a cycle with an increase in the number of cycles was associated with increasing dislocation yield stress (Figure 4). With an increase in the dislocation yield stress at 0.5% strain, the reversible elastic strain area increased, which led to a decrease in the residual strain in the cycle. Accordingly, the value of the accumulated strain in the case of the RHT regime was the largest (5.4%) compared to the TMT regimes (Figure 6).

The values of the dislocation yield stress before the start of mechanocycling for the RHT regime are the lowest (unhardened state). After TMT, due to structural and substructural hardening, the elastic limit increases; accordingly, the accumulated strain in the cycle is less.

**Figure 7:**
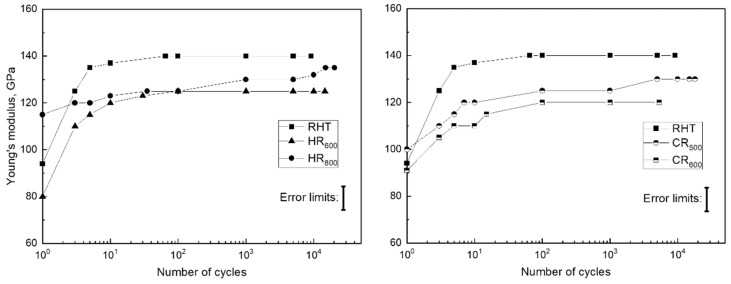
Evolution of Young’s modulus during mechanocycling of the Fe-30Mn-5Si alloy after various treatment regimes.

The values of Young’s modulus, determined by the load branch, are shown in Figure 7. It can be seen that Young’s modulus increases in the first cycles for all TMT regimes and then get stable. Such Young’s modulus behavior during mechanocycling was already observed in [38] for Ti-Zr-Nb SMA. This is because the SMA elastic modulus measured in such mechanical tests is an apparent value not only due to the method specificity but also due to the structural behavior features of these alloys under stress. In this case, first martensite crystals appear at stresses significantly below the dislocation yield stress [23,39], resulting in the downward inclination of the strain–stress diagram ascending branch and underestimating the elastic modulus. With the strengthening during mechanocycling, the stress for the martensite formation start raises, and the slope of the ascending branch increases.

At the same time, it should be noted that the hardened state after TMT demonstrates a slightly lower steady-state value of Young’s modulus resulting from mechanocycling. This can be explained by the lower *M_s_* temperature, which is closer to the test temperature (room temperature) since it is known that TMT leads to a decrease in the reversible martensitic transformation points where the premartensitic lattice softening effect takes place [23,24,39].

The number of cycles to failure (*N_max_*) for each investigated treatment regime is listed in Table 1. It was found that after all TMT (except CR_600_), the alloy exhibited a much larger *N_max_* than after RHT.

The total *N_max_*, in general, correlates with the value of the dislocation yield stress. As mentioned above, the higher the value of the dislocation yield stress, the less the irreversible plastic strain is included in the deformation process.

It should be noted that the maximum *N_max_* value exhibited by the HR_800_ sample can be caused by the much smaller grain size (100 μm) compared to other regimes providing the grain size of about 500 μm (see Section 3.1). The decrease in grain size leads to increased grain boundary concentration, enhancing their barrier effect on the dislocation yield. In this case, the *N_max_* of the HR_800_ regime can be explained by the higher dislocation yield stress due to the smallest grain size, following the Hall–Petch law.

The obtained superiority of TMT over the conventional heat treatment in the functional fatigue parameters was observed in previous studies of some other SMA systems, such as Ti-Ni [40,41], Ti-Nb-Zr [42,43], and Ti-Zr-Nb [44] as well.

### 3.3. Effect of Mechanical Cycling on the Phase Composition of the Fe-30Mn-5Si Alloy

Following [24], only the HR_800_ regime results in the austenite grain refinement compared to other regimes. In this regard, the HR_800_ regime was chosen for this part of the study to eliminate the coarse-grained structure effect on the XRD analysis results.

**Figure 8:**
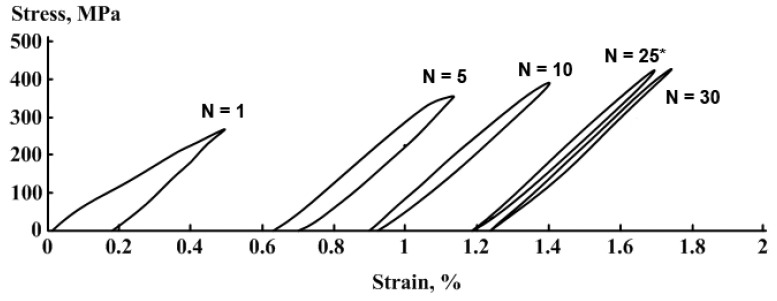
Stress–strain diagrams at *N^th^* loading–unloading cycle for the Fe-30Mn-5Si alloy after HR_800_ (*—the beginning of the loop closure).

Stress–strain diagrams obtained after a various number of loading–unloading cycles are presented in Figure 8. An initial range of each stress–strain diagram demonstrates a linear stress increase. Then, after reaching a dislocation yield stress, deviation from linearity began, which increased with the strain increase and development of a dislocation flow mechanism. Note that the similar deviation can be attributed to the intensification of the stress-induced *γ*→*ε* transformation. That is confirmed by the results of [25,45,46,47,48], which showed that after reaching the apparent dislocation yield stress, the deformation process included the *γ*→*ε* martensitic transformation.

**Figure 9:**
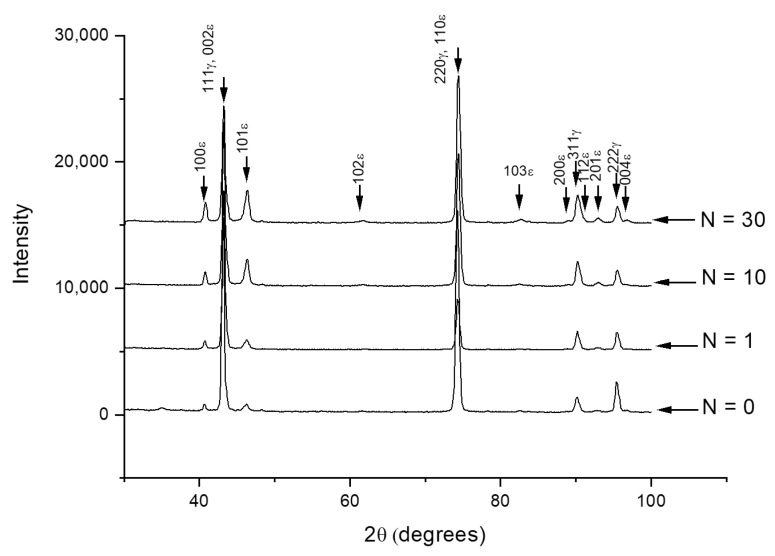
X-ray diffractograms Fe-30Mn-5Si alloy (HR_800_) after various number of loading–unloading cycles (*N*) in accordance with Figure 8.

Figure 9 presents X-ray diffractograms of HR_800_ samples obtained after cyclic loading–unloading with various number of cycles up to *N* = 30 in accordance with diagrams presented in Figure 8. Figure 9 shows that before cycling (*N* = 0), the sample contains both *γ*-austenite and *ε*-martensite. Note that the mechanocycling is carried out at room temperature while the starting temperature *M_s_* for the *γ*→*ε* transformation locates at 38 °С while its finishing temperature *M_f_*, in a subzero temperature range [24]. Consequently, the alloy must be in a two-phase *γ* + *ε* state at room temperature after HR_800_ treatment. Therefore, during the mechanocycling, additional portions of the stress-induced martensite could form from the retained austenite [25], and this new martensite must be stable because the starting temperature for the reverse *ε*→*γ* transformation is as high as *A_s_* = 136 °С [24].

The intensities of the XRD lines of *γ*- and *ε*-phases in the diffractograms change with the growing number of cycles (Figure 9). The visible lines in the diffractogram were formed by overlapping pairs of *γ*- and *ε*-phase reflexes, as follows: 111_γ_ and 002_ε_, 220_γ_ and 110_ε_, and 311_γ_ and 112_ε_, and therefore, they cannot be used for evaluation of phase composition changes. For this purpose, seven lines of the *ε*-phase and one line of the *γ*-phase can be involved, as follows: 100_ε_, 101_ε_,102_ε_, 103_ε_, 200_ε_, 201_ε_, 004_ε_, and 222_γ_. The accuracy of this evaluation is relatively low; nevertheless, the large number of free martensite lines allows a confident quantity evaluation.

**Figure 10:**
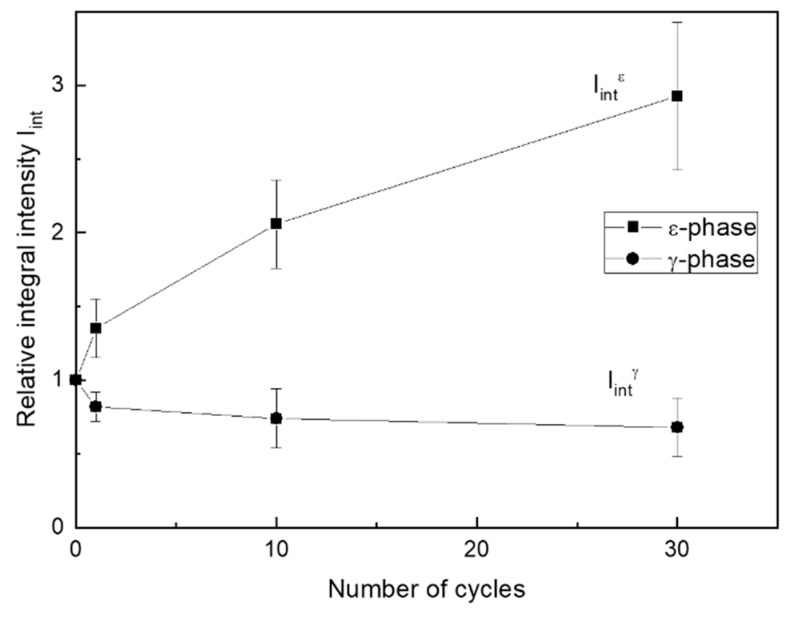
Changes in the relative integral intensity of X-ray lines during mechanocycling. Fe-30Mn-5Si alloy, HR_800_. I_int_^ε^—summarized intensities of *ε*-phase lines; I_int_^γ^—intensity of 222_γ_ line.

Figure 10 shows relative variation of summarized integral intensity of all free *ε*-martensite lines and 222_γ_ line during cycling up to *N* = 30. The intensity summarizing of the martensite lines aims to consider a possible non-uniformity of their changes resulting from martensite reorientation under stress.

As follows from Figure 9 and Figure 10, initial loading–unloading cycles resulted in a significant *γ*-austenite fraction decrease and *ε*-martensite fraction increase. Note that the growth of the accumulated strain was almost completed at 25th cycle (Figure 8). Considering the XRD data, such mechanical behavior indicates that this strain accumulated at least partially due to the formation of a stable *ε*-martensite, which did not disappear after unloading. The apparent dislocation yield stress increased during mechanocycling due to an increase in dislocation density and martensite amount, and after reaching *N* = 25 cycles, it exceeded the maximum stress reached at 0.5% strain. Therefore, the ongoing deformation occurred in the elastic zone. The possibility of the superelasticity affects partial manifestation due to a reverse reorientation of martensite or even reverse transformation during unloading can also be assumed. However, in situ XRD experiment during loading–unloading must be performed to verify such possibility.

Changes of the *γ*- and *ε*-phase volume fractions can be evaluated from Figure 10, which shows that after 30 cycles, the intensity of martensite lines increased approximately threefold, while that of austenite decreases by half. A simple calculation shows that the corresponding quantitative ratio of these phases changed from 20/80 to 60/40.

To estimate the lattice imperfectness (dislocation density) changes during mechanocycling, a half-height width of strong X-ray lines 111_γ_, 002_ε_ and 220_γ_, 110_ε_ was measured (Table 2). Such estimation is admissible because the martensite inherits the dislocation substructure of the parent austenite [39]. Table 2 shows that the line width somewhat increases as a result of mechanocycling. This indicates an increase of lattice distortions caused by the dislocation density rise, i.e., transformation-induced hardening introduced during *γ*→*ε* transformation and reorientation of the existing *ε*-martensite [49].

### 3.4. Corrosion Fatigue Bending Tests in Hanks’ Solution

**Figure 11:**
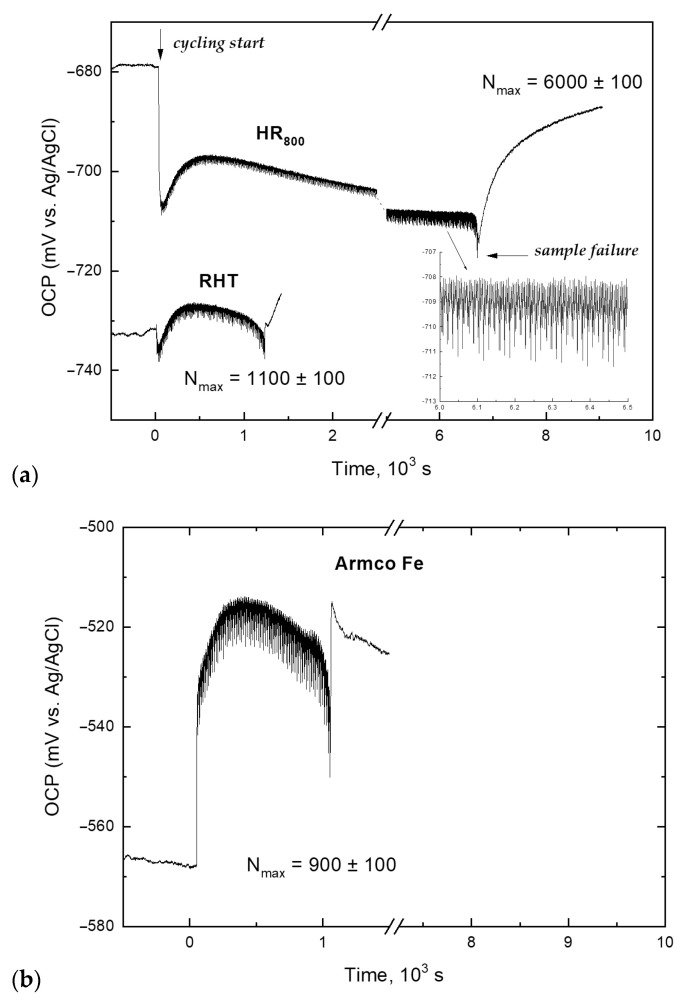
Open-circuit potential curves of alloy samples Fe-30Mn-5Si (**a**) and Armco iron (**b**) in the process of mechanocycling in Hanks’ solution. Arrows show the time points of cycling start and sample failure in (**a**) as an example.

Figure 11 shows typical OCP curves of the Fe-30Mn-5Si alloy (RHT and HR_800_ regimes) during cyclic dynamic loading in Hanks’ solution at 37 °С; for comparison, the OCP curve of Armco’s iron is also presented. It can be seen from Figure 11a, that the moment of loading start features the OCP shifted to more negative values (by 10–30 mV), regardless of the treatment regime. This is caused by the disruption of the corrosion product layer formed during prior exposure to the test solution.

According to XPS data, the surface layer consists of FeOOH (bond energy 710.2 eV), MnO (640.2 eV), and a trace amount of Si in an intermediate oxidation state (pure Si—99.3 eV and SiO_2_—103.3 eV). The distribution of corrosion products on the alloy surface is inhomogeneous, with the Mn/Fe atomic concentration ratio in the surface layer exceeding 1, which confirms the high electrochemical activity of manganese.

A freshly formed local area of the alloy surface is a potential zone for corrosion-fatigue crack initiation and propagation since the metal of the renewed surface is spent on corrosion product formation. The breakdown of the corroded layer uniformity occurs in the loading half-cycle; therefore, in the process of further cycling, the effective cross-section of the sample decreases, which causes an increase in the specific load in the zone of local dissolution of alloy components.

During the subsequent cycling, a gradual potential increase within several millivolts is observed, accompanied by its fluctuations with a frequency equal to the frequency of loading cycles (0.9 Hz). As can be seen from Figure 11a, in contrast to the RHT mode, the HR_800_ treatment did not lead to an excess of the OCP value before cycling; moreover, even the initial value was not reached. In both cases, after a certain number of cycles, there was a steady tendency towards OCP lowering until the sample failure. In this case, the time to failure of the HR_800_ samples was five times longer than that for the RHT regime. Further exposure features spontaneous anodic polarization due to the dissolution process inhibition as the fracture region of the sample gets filled with corrosion products.

As follows from Section 3.3, the mechanocycling process is accompanied by the yield stress increase caused by structural and substructural hardening due to an increase in the dislocation density. Plastic deformation changes the physical and mechanical properties of the surface and affects its ability to adsorb anions from a corrosive medium, which is associated with an increase in the “anodic” state of the surface. This mechanochemical effect is equivalent to additional anodic polarization, which is observed immediately after the initial OCP drop after the load is switched on (indicated by the arrow in Figure 11a). In addition, the dynamic load facilitates an enhanced convective oxygen supply from the medium to the alloy surface. Oxygen is known as an effective cathodic depolarizer for metals corroding in neutral media in an active state; its facilitated delivery to the cathode regions of the alloy surface assists the cathodic depolarization reaction (О_2_ + 2Н_2_О + 4е = 4ОН^−^), and consequently accelerates the corrosion process [50].

In turn, the irreversible corrosion interaction of the wrought alloy with the medium (chemomechanical effect) facilitates the process of rearrangement of interatomic bonds in the surface layer, which also contributes to the plasticization of the metal [51].

According to XRD analysis, the alloy has a two-phase structure, which is characterized by a certain volume ratio of *γ*-austenite and *ε*-martensite depending on the treatment regime. Upon contact with a corrosive medium, these structural components form a galvanic pair, in which austenite is the cathode and martensite is the anode [17,24]. The accumulation of plastic deformation during mechanocycling is accompanied by an irreversible *γ→ε* transformation, i.e., each loading cycle causes an increase in the volume fraction of the anodic component of the structure (*ε*-phase).

Thus, the kinetics of the OCP changes during mechanocycling is influenced by two competing processes—mechanical activation, which enhances the adsorption capacity for the corrosion product formation on the freshly formed surface, and the increase in the fraction of a more electronegative *ε*-phase, which is accompanied by a monotonic OCP shift to the negative direction after a certain number of cycles.

It is essential that achieving the yield stress constant value for RHT samples with 1% deformation requires twice as many loading cycles than for the HR_800_ regime (Section 3.2). This allows assuming that the reason for the observed OCP change kinetics for the RHT samples is the predominance of the more electropositive *γ*-phase in the alloy structure. The result obtained agrees with the results of [17,52], according to which martensite crystals are efficient mechanical barriers to fatigue crack propagation, which is confirmed by the earlier fracture of samples after RHT due to the lower volume fraction of *ε*-phase crystals.

The barrier role of martensite in crack propagation is also indicated by the results of corrosion fatigue behavior of Armco iron (Figure 11b), which structure consists of α-ferrite (anodic phase) and a small amount of cementite (Fe_3_C or Fe_23_C_6_-cathodic phase [17,50]). The absence of phase transformation during mechanocycling determines the difference in the observed OCP change kinetics and the number of cycles to failure. Since the anodic structural component occupies almost the entire volume of the material, the predominant factor here is not the local destruction of the corrosion product layer but the high anodic efficiency of the renewed surface after activation by the load impact. This significantly enhances the surface’s ability to adsorb the oxidizing components of the corrosive medium, which promotes the accelerated formation of corrosion products that inhibit the ionization of the alloy components and cause instantaneous anodic polarization. Further slight OCP growth and its subsequent lowering are also accompanied by small fluctuations. However, they are not associated with phase transformations but reflect the surface activation during the loading half-period and the formation of products of interaction with the corrosive medium during the unloading half-cycle. The recovery of the oxidized surface destroyed area requires the consumption of metal. With each subsequent loading cycle, the specific load in the zone of crack propagation increases, which, in the absence of obstacles to its growth, causes accelerated fracture of the specimen.

### 3.5. Temperature Dependence of Young’s Modulus

**Figure 12:**
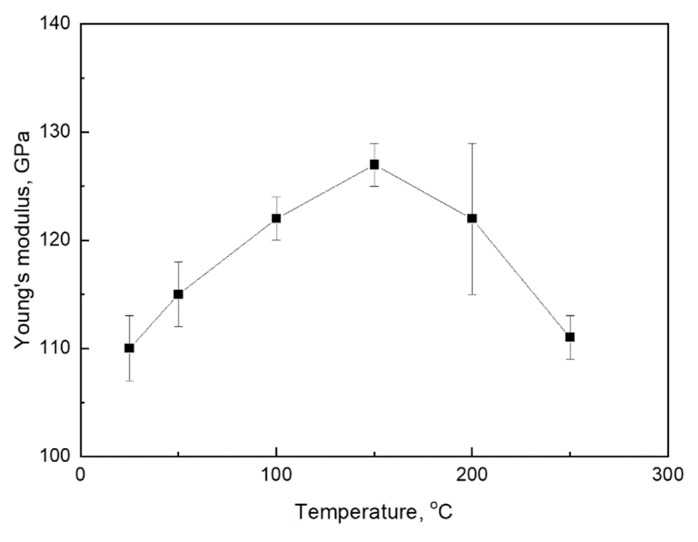
Temperature dependence of Young’s modulus of the Fe-30Mn-5Si alloy (HR_800_).

One of the essential criteria for using the Fe-30Mn-5Si alloy as a material for medical bone implants is the low Young’s modulus value. The previous study shows that TMT involving HR_800_ regime leads to a significant lowering of the *M_s_* temperature towards a human body temperature (37 °C) compared to RHT (75 °C) [24]. It was considered as the advantage that allows obtaining the Young’s modulus minimum value near human body temperature, since in many SMAs Young’s modulus decrease due to the development of premartensitic “lattice softening” upon the test temperature approaching the *M_s_* point takes place [23,39]. To verify this trend for the Fe-Mn-Si system, the change in Young’s modulus of the Fe-30Mn-5Si alloy at different test temperatures was studied. The apparent Young’s modulus *E* was measured as a tg*α*, where *α* is a slope angle of the linear loading curve to the strain axis. The results are summarized in Figure 12.

At 250 °C, Young’s modulus is *E* = 111 ± 3 GPa; the test temperature lowering leads to an increase in Young’s modulus up to 128 ± 2 GPa at 150 °C. This is a “normal” temperature dependence of the elastic modulus underlied by a decrease in the equilibrium interatomic distance and an increase in the interatomic bond strength due to thermal compression with decreasing temperature. A further test temperature lowering from 150 °C to room temperature, however, is accompanied by an “anomalous” Young’s modulus decrease and its minimization (110 ± 3 GPa) at *M_s_* temperature. This trend reflects the crystal lattice premartensitic “softening”, which is attributable to SMAs. Consequently, this phenomenon is proved for the Fe-30Mn-5Si alloy as well.

## 4. Conclusions

(1) A comparative TEM study of the Fe-30Mn-5Si (wt %) alloy performed after a reference heat treatment (RHT) and thermomechanical treatments (TMT) comprising hot rolling at 600 and 800 °C (HR_600_ and HR_800_, respectively) followed by water cooling, reveals a two-phase mixture of FCC *γ*-austenite and HCP *ε*-martensite. The *ε*-phase amount after RHT was significantly higher than after TMTs due to incompleteness of *γ*→*ε* transformation on cooling after TMTs. The *γ*-austenite matrix and *ε*-martensite crystals did not contain high dislocation density. A well-developed dislocation substructure formed in *γ*- and *ε*-phases due to dynamic recrystallization of austenite during hot deformation at 800 °C. The most highly dislocated substructure formed after HR_600_; it consisted of dislocation tangles and cells, while a typical polygonized dislocation substructure consisting of perfect subgrains did not form.
(2) As a result of functional tensile fatigue tests with 0.5% strain in a cycle after RHT and all TMTs, the changes of stress–strain diagrams parameters were as follows: the increase of dislocation yield stress, accumulated strain, and apparent Young’s modulus, and decrease of residual strain in a cycle, and their subsequent stabilization after 10–65 cycles. After TMTs, the residual strain in a cycle dropped to almost zero much faster than after RHT; the accumulated strain was much larger while the stabilized yield stress was lower, and Young’s modulus was significantly lower. Based on the XRD study carried out in a cycling range until the parameters’ stabilization, these parameters’ changes were explained in terms of a decelerated increase in dislocation density and amount of *ε*-martensite formed during the stress-induced *γ*→*ε* transformation in that range. The number of cycles to failure after TMTs comprising hot rolling was much higher than after RHT and reached its maximum of about 20,000 after the HR_800_ regime.
(3) A specific temperature dependence of the apparent modulus *E* was determined by comparative tensile testing and DSC study in a temperature range of 250–20 °C. The specificity features *E* decreased instead of its normal increase with decreasing temperature as the *M_s_* temperature was approached. For the HR_800_ regime, E dropped from 130 GPa at 150 °C to its minimum of 110 GPa at room temperature, i.e., it became equal to the elastic modulus of pure titanium conventionally used for bone implants. Thus, the applicability of this approach to the Fe-30Mn-5Si SMA was confirmed, which comprises increasing its biomechanical compatibility by maximizing the convergence of the *M_s_* temperature and the operating temperature (37 °C) due to the lattice premartensitic softening phenomenon.
(4) Based on the analysis of the open-circuit potential (OCP) measurement during immersion of the alloy’s RHT and HR_800_ samples in Hanks’ solution at 37 °C, a non-monotonic nature of the OCP change after the initial drop at the loading action start is revealed and explained, as follows: (1) mechanical activation enhanced the tendency of the newly formed surface to adsorb anions and form corrosion products, resulting in anodic polarization; (2) the irreversible stress-induced martensitic *γ*→*ε* transformation resulted in an increase in the volume fraction of the more electronegative *ε*-phase in each loading half-cycle, which, after reaching a certain number of cycles, manifests itself in a stable shift of the samples’ potential to more negative values. The accumulation of the martensitic ε-phase is the reason for the high corrosion fatigue resistance after TMT using the HR_800_ regime, which is associated with the efficient barrier effect of martensite crystals that inhibits the propagation of fatigue cracks, with this effect being supported by much higher yield stress evidencing more developed dislocation substructure as compared to the RHT.

## Figures and Tables

**Table 1:** The number of cycles to failure for the Fe-30Mn-5Si alloy.

Number of Cycles to Failure	RHT	HR_600_	HR_800_	CR_500_	CR_600_
*N_max_*	9139	14,642	20,330	17,984	5347

**Table 2:** Evolution of X-ray line half-height width during mechanocycling.

Number of Cycles	111_γ_, 002_ε_, *2θ* deg.	220_γ_, 110_ε_, *2θ* deg.
*N* = 0	0.41	0.53
*N* = 1	0.41	0.53
*N* = 10	0.41	0.54
*N* = 30	0.45	0.58

## Data Availability

Data sharing is not applicable to this article.

## Appendix A. Abbreviations and Symbols

SMA	shape memory alloy;
*γ*	austenite phase with face-centered cubic (FCC) crystal lattice;
*ε*	martensite phase with hexagonal close-packed (HCP) crystal lattice;
*M_s_*	start temperature of the *γ→ε* forward martensitic transformation;
*M_f_*	finish temperature of the *γ→ε* forward martensitic transformation;
*A_s_*	start temperature of the *ε→γ* reverse martensitic transformation;
*A_f_*	finish temperature of the *ε→γ* reverse martensitic transformation;
TMT	thermomechanical treatment;
RHT	reference heat treatment;
HR	hot rolling;
CR	cold rolling;
XRD	X-ray diffraction;
2*θ*	X-ray diffraction angle;
TEM	transmission electron microscopy;
BF	bright field;
DR	dark field;
SAED	selected area electron diffraction;
XPS	X-ray photoelectron spectroscopy;
*E*	Young’s modulus;
*σ*_0.05_	dislocation yield stress;
*ε_f_*	residual strain in a mechanical cycle;
*ε_acc_*	accumulated strain after each mechanical cycle;
*N_max_*	number of cycles to failure;
OCP	open circuit potential.
